# A Novel Cell-Based Model for a Rare Disease: The Tks4-KO Human Embryonic Stem Cell Line as a Frank-Ter Haar Syndrome Model System

**DOI:** 10.3390/ijms23158803

**Published:** 2022-08-08

**Authors:** Loretta László, Hédi Maczelka, Tamás Takács, Anita Kurilla, Álmos Tilajka, László Buday, Virag Vas, Ágota Apáti

**Affiliations:** 1 Institute of Enzymology, Research Centre for Natural Sciences, 1117 Budapest, Hungary; 2 National Laboratory for Drug Research and Development, 1117 Budapest, Hungary; 3 Doctoral School of Biology, Institute of Biology, ELTE Eötvös Loránd University, 1117 Budapest, Hungary; 4 Basic and Translational Medicine Doctoral School, Semmelweis University, 1085 Budapest, Hungary

**Keywords:** FTHS, Frank-Ter Haar syndrome, CRISPR-Cas9, Tks4, human embryonic stem cells, cell commitment, mesenchymal stem cells

## Abstract

Tyrosine kinase substrate with four SH3 domains (Tks4) scaffold protein plays roles in cell migration and podosome formation and regulates systemic mechanisms such as adult bone homeostasis and adipogenesis. Mutations in the Tks4 gene (*SH3PXD2b*) cause a rare developmental disorder called Frank-Ter Haar syndrome (FTHS), which leads to heart abnormalities, bone tissue defects, and reduced adiposity. We aimed to produce a human stem cell-based in vitro FTHS model system to study the effects of the loss of the Tks4 protein in different cell lineages and the accompanying effects on the cell signalome. To this end, we used CRISPR/Cas9 (clustered, regularly interspaced, short palindromic repeats (CRISPR)/CRISPR associated (Cas9)) to knock out the *SH3PXD2b* gene in the HUES9 human embryonic stem cell line (hESC), and we obtained stable homo- and heterozygous knock out clones for use in studying the potential regulatory roles of Tks4 protein in embryonic stem cell biology. Based on pluripotency marker measurements and spontaneous differentiation capacity assays, we concluded that the newly generated Tks4-KO HUES9 cells retained their embryonic stem cell characteristics. We propose that the Tks4-KO HUES9 cells could serve as a tool for further cell differentiation studies to investigate the involvement of Tks4 in the complex disorder FTHS. Moreover, we successfully differentiated all of the clones into mesenchymal stem cells (MSCs). The derived MSC cultures showed mesenchymal morphology and expressed MSC markers, although the expression levels of mesodermal and osteogenic marker genes were reduced, and several EMT (epithelial mesenchymal transition)-related features were altered in the Tks4-KO MSCs. Our results suggest that the loss of Tks4 leads to FTHS by altering cell lineage differentiation and cell maturation processes, rather than by regulating embryonic stem cell potential.

## 1. Introduction

Tks4 is a scaffold protein that functions as a hub-protein for signalling molecules. Tks4 is involved in EGF-signalling as the EGFR-activated Src kinase phosphorylates Tks4 [1,2]. The role of Tks4 in podosome formation, and thus involvement of cancer invasion has already been described; for example, Tks4 binds several podosome-organizing proteins, including the actin filament regulator cortactin and extracellular matrix-degrading MT1-MMP [2,3]. Tks4 is also necessary for bone marrow MSC (mesenchymal stem cells) differentiation into osteocyte and adipocyte lineages in vitro and for proper bone architecture formation and white/brown adipocyte homeostasis in vivo in mice [4,5,6]. Although several aspects of the physiological roles of Tks4 have already been revealed, the functional role of Tks4 in human embryonic development and the mechanism by which loss of Tks4 influences the complex symptoms associated with Frank-Ter Haar syndrome (FTHS, OMIM:249420) remain unknown. FTHS is a severe developmental birth defect characterized by a complex collection of phenotypes, including bone deformities (craniofacial malformations with wide fontanel and abnormal toothing, kyphosis, shortened bended bones), heart developmental defects, early onset glaucoma, reduced adipose tissue, and infertility [7,8,9,10,11]. Remarkably, FTHS severely affects skeletal bone and adipose tissue composition, all of which originate from MSCs, suggesting that MSC differentiation is disturbed during development in affected individuals.

Iqbal and colleagues traced the genetic backgrounds of several FTHS-affected families and revealed that mutations in both alleles of the *SH3PXD2b* gene (leading to loss of Tks4 protein) were present in disease-affected individuals [12]. Indeed, the majority of FTHS babies born to consanguineous parents have the same inherited mutation in both *SH3PXD2b* alleles. FTHS shows an autosomal-recessive mode of inheritance in which the parents carry early STOP codon in *SH3PXD2B* on chromosome 5q35.1. After the identification of the genetic features underlying the FTHS phenotypes, studies were initiated to describe the effect of the mutant Tks4 protein in mouse models. Bushman et al. demonstrated that the Tks4 mRNA level is high in wild type (WT) mouse embryos, and that Tks4-deficient mice (Tks4-KO) are born with severe multiorgan phenotypes, similar to the symptoms of FTHS in humans [13]. Parallel with this study, an abundance of Tks4 mRNA/protein in human embryonic tissues was also demonstrated in the EMBL-EBI-Expression Atlas database [14,15,16].

As there is no human in vitro model for FTHS, we aimed to develop a human cell-based model system to study this complex disease. Human pluripotent stem cell lines (hPSC) can differentiate into almost every cell type found in the human body, thereby providing an excellent opportunity to study human developmental processes as well as a flexible tool for disease modelling. In addition to their vast differentiation capability, hPSCs have theoretically unlimited self-renewal and proliferative capacity, making them ideal for genetic engineering.

The CRISPR/Cas9 (clustered, regularly interspaced, short palindromic repeats (CRISPR)/CRISPR associated (Cas)) system has rapidly become a powerful genome editing tool given its highly precise and efficient targeting method and relatively simple implementation. This molecular genetic system is remarkably powerful for modelling genetic disorders in vitro via the generation of isogenic hPSC clones that differ from the WT hPSCs in only the gene of interest [17,18]. In our previous work, we used CRISPR/Cas9 to generate *SH3PXD2b* knock out cancer cell lines [19]. This cell line is derived from a colon carcinoma (HCT116) carrying several cancer-prone mutations, e.g., KRAS (G13C) and PIK3CA. This cell line, thus, is suitable for testing Tks4 effects during cancer development and is not an FTHS model cell line for studying Tks4 in embryonic cell biology. Here, we report that we have used the same Cas9-guide combination to generate two *SH3PXD2b* knock out human embryonic stem cell line (HUES9) clones, thereby creating a cell-based model system to study FTHS. HUES9 is a widely used human blastocysts-derived embryonic stem cell line having the advantage of relatively easy handling via robust clonal expansion and efficient electroporation-based gene targeting without the loss of pluripotent characteristics (such as board differentiation potential) or karyotypic changes. In this study using the newly generated Tks4-KO HUES9 cell lines, we describe the effects of Tks4 loss on stem cell properties in via spontaneous cell differentiation. Furthermore, we have directed the Tks4-KO HUES9 cells to differentiate into MSCs and followed the processes by measuring key differentiation markers via a protein array.

## 2. Results

### 2.1. Generation of Tks4 Knockout HUES9 Cell Lines

**Figure 1:**
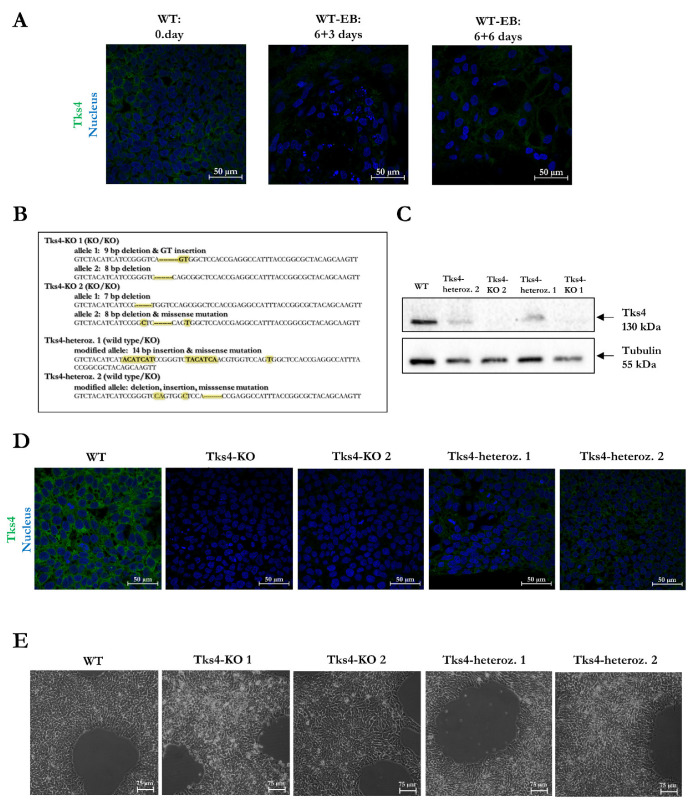
Generation of Tks4-knockout HUES9 cells. (**A**) Representative confocal images showing the typical immunostaining for Tks4 during spontaneous differentiation via EB formation in HUES9 cells; (**B**) Sequence of exon 2 of *SH3PXD2b* in the two newly generated homozygous-mutated (KO/KO) clones and the two heterozygous clones (WT/KO). The mutant nucleotides are labelled with yellow marker in the region of interest of the sequenced allele. Validation of Tks4 loss at the protein level in the two Tks4-KO HUES9 clones in comparison with the original HUES9 and heterozygous cells using (**C**) western blotting and (**D**) immunocytochemistry (ICC): Tks4—green, nucleus—blue (DAPI); (**E**) Representative bright field images showing that all of the cell lines retain human ESC morphology (scale bar 75 µm).

The presence and consequently the potential involvement of Tks4 in human embryonic development has been previously demonstrated in the Expression Atlas [14]; therefore, we suspected that human embryonic stem cell line (hESC) lines also express Tks4. To shed light to the role of Tks4 in embryonic stem cell biology, we selected a hESC line to assess the roles of Tks4 in vitro. First, we tested the HUES9 cells for the presence of Tks4 to assess the usefulness of this cell line for creating Tks4-KO embryonic stem cells. An immunocytochemistry (ICC) analysis showed that HUES9 cells express Tks4 protein and that Tks4 was present during spontaneous differentiation (Figure 1A). Therefore, we used HUES9 cells to investigate the role of Tks4 in human embryonic stem cells by knocking out its gene (*SH3PXD2b*) using the CRISPR/Cas9 system. We induced mutations in exon 2 of *SH3PXD2b* and confirmed the genetic alterations via sequencing (Figure 1B). Two Tks4-KO HUES9 clones were produced where *SH3PXD2b* knockout was achieved via insertion-deletion events that led to the introduction of stop codons in both alleles. In addition to the homozygous Tks4-KO clones, two *SH3PXD2b* -mutated heterozygous HUES9 clones were also generated. In the heterozygous HUES9 clones, sequence analyses revealed an insertion-deletion mutation next to the Cas9 cut site in one allele, while the other allele remained intact. The absence of Tks4 protein in the Tks4-KO HUES9 clones and the presence of Tks4 protein in the heterozygous-mutated clones were confirmed via western blot analysis and fluorescence immunocytochemistry (Figure 1C,D). All of the HUES9 clones were also shown to have a normal karyotype (Supplementary Figure S1A).

### 2.2. Validation of the Pluripotency of the Tks4-KO HUES9 Cells

**Figure 2:**
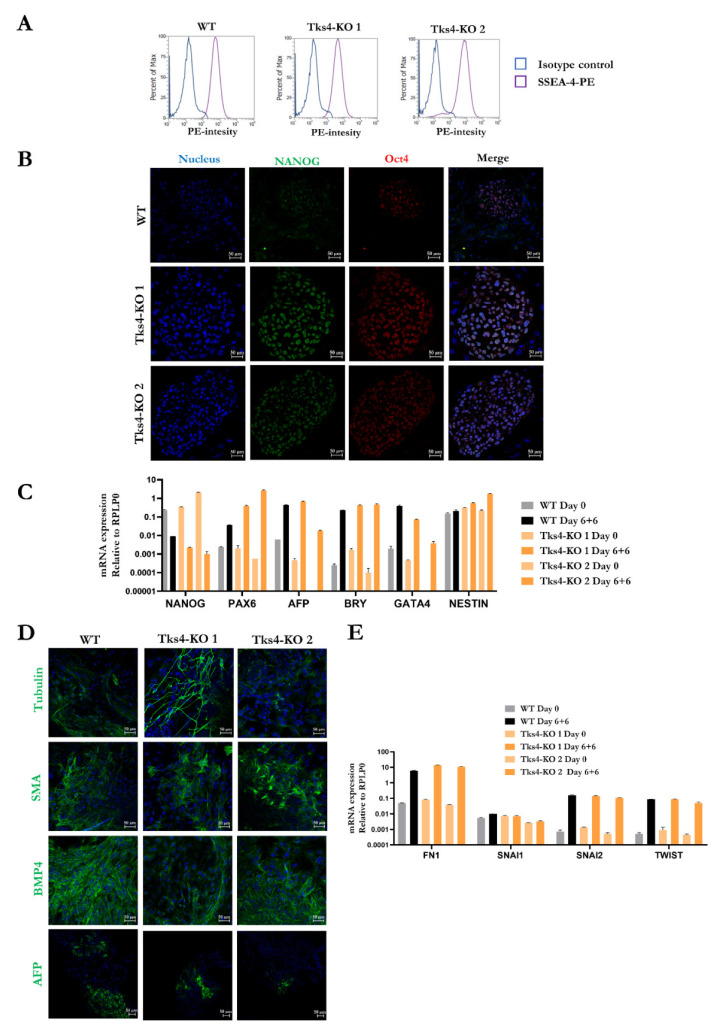
Assessment of the pluripotency of the Tks4-KO HUES9 cells. (**A**) The SSEA4 expression levels in WT cells and the Tks4-KO cells were analysed via flow cytometry. Isotype controls were used as a negative control on each plot; (**B**) Immunofluorescent staining showing the Oct4 (red) and Nanog (green) expression levels in undifferentiated cells; (**C**) The expression levels of the pluripotency-related genes Nanog and differentiation-related genes Pax6, alpha fetoprotein (AFP), brachyury (BRY), GATA4, and NESTIN were analysed via RT-qPCR to assess the efficiency of spontaneous differentiation. Gene expression was normalized to the value of RPLP0; (**D**) The differentiation potential into all the three germ layers of WT cells and the Tks4-KO cells were assessed via ICC of β-III-tubulin, smooth muscle actin (SMA), bone morphogenetic protein 4 (BMP4), and AFP after 6 + 6 days of the spontaneous differentiation. Nuclei were counterstained with DAPI (blue); (**E**) Expression levels of EMT-related markers (Fibronectin (mRNA:FN1), Snail1 (mRNA:SNAI1), Snail2 (mRNA:SNAI2), Twist (mRNA:TWIST)) were measured via RT-qPCR and compared to those of the original stem cell lines (day 0) within the spontaneous differentiated derivatives (day 6 + 6 of differentiation).

The loss of Tks4 does not induce major morphological changes in HUES9 cells and does not alter the proliferative capacity of the Tks4-KO mutant clones (Figure 1E and Figure S1B). Next, we wondered if mutation of Tks4 would lead to altered pluripotency. All of the mutant clones showed more than 95% positivity for the SSEA4 pluripotency marker as detected via flow cytometry and were positive for the Nanog and Oct4 pluripotency markers as demonstrated via immunocytochemistry (Figure 2A,B). The Tks4-KO HUES9 clones showed similar pluripotent phenotypes as the WT cells. We obtained similar results for the heterozygous clones (Supplementary Figure S2A,B).

We have also examined the spontaneous differentiation potential of the generated Tks4-KO cells using the embryoid body formation method. Pluripotent stem cells were maintained on mouse fibroblast cells to form colonies for 2 passages and then plated onto ultra-low attachment plates for 6 days to form embryoid bodies (EB). All of the studied cell lines formed EBs. On day 7, EBs were seeded on a gelatine coated surface for another 6 days. To follow the success of the spontaneous differentiation process in the cell cultures, we used RT-qPCR to detect the down regulation of the pluripotency marker Nanog and the upregulation of several germ layer markers (ectoderm-Pax6 and Nestin, mesoderm-Brachyury and GATA4, endoderm-AFP). As expected, Nanog expression decreased and the expression levels of the differentiation markers increased in differentiated cultures (Figure 2C). The heterozygous differentiated clones also showed decreased Nanog expression, and they also expressed all of the examined differentiation markers in differentiated samples (Supplementary Figure S2C). To detect the presence of cells from the three germ layers among the differentiated cell population, we stained the fixed samples on day 12 for β-III-tubulin (a marker for ectoderm differentiation), SMA (a mesoderm marker), AFP (an endoderm marker), and BMP4 (a mes/endoderm marker) (Figure 2D and Figure S2D). The results of the immunocytochemistry analyses showed that each germ layer marker was present in the Tks4-KO HUES9 clones similar to the parental cells, except for Tks4-KO 2 clone where AFP protein was present at a lower level. Our results indicate that lack of Tks4 does not interfere with basic germ layer formation.

In our previous study, we discovered that lack of Tks4 in a colon cancer cell line results in altered cell phenotypes and induces an epithelial to mesenchymal transition (EMT)-like process in Tks4-KO HCT116 cells. To investigate whether lack of Tks4 influences EMT not only in cancer cells but also in embryonic stem cells, we measured the expression levels of the Fibronectin, Snal1, and Twist EMT markers (Figure 2E and Figure S2E). The results showed that during the spontaneous differentiation process the expression levels of these markers increased dramatically; however, we did not detect expression level differences between WT cells and the Tks4-KO embryonic stem cell clones.

### 2.3. Directing the Differentiation of the Tks4-KO HUES9 Cells into Mesenchymal Stem Cells (MSCs)

**Figure 3:**
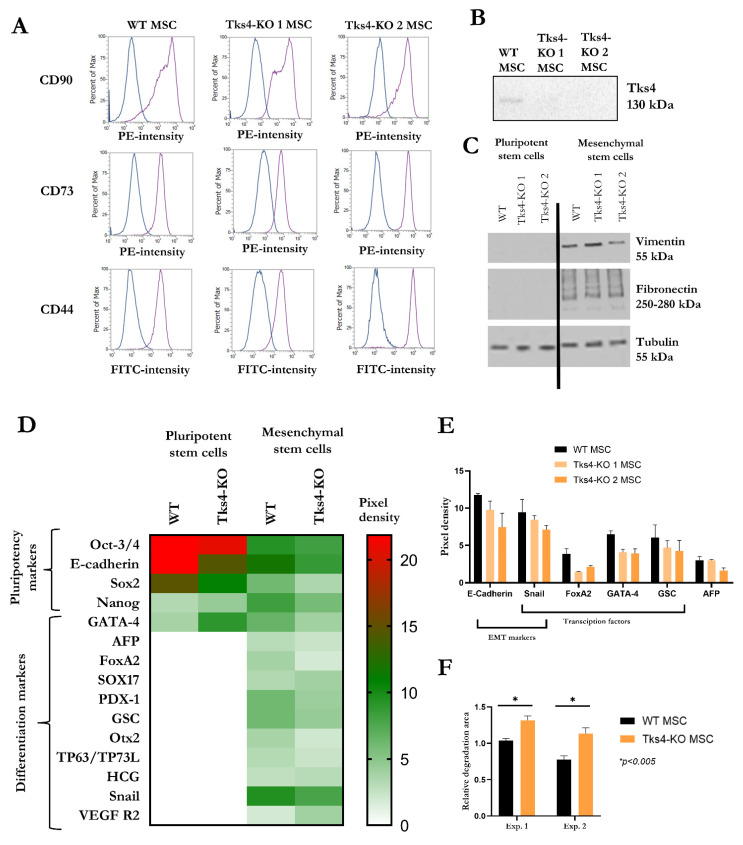
(**A**) Representative flow cytometry histograms showing CD90, CD72, and CD44 staining of WT (WT) MSC and the two Tks4-KO MSC clones. (Blue lines represent the isotype control labelling); (**B**) Representative western blot showing the presence of Tks4 in WT MSC cells and the lack of Tks4 protein in Tks4-KO MSC clones; (**C**) Western blot analysis of the EMT (and differentiation) markers Vimentin and Fibronectin in pluripotent stem cells (WT and Tks4-KO) and the generated MSC (WT and Tks4-KO) lines; (**D**) Heatmap comparing the proteome profiler stem cell array results of the pluripotent stem cells (WT and Tks4-KO) and the generated MSC (WT and Tks4-KO) lines. The two Tks4-KO clones were measured separately but the obtained data sets were combined and the averages of two independent biological samples were presented; (**E**) Protein levels of early differentiation markers (E-cadherin, Snail, FoxA2, GATA4, goosecoid (GSC), AFP) measured in WT and Tks4-KO MSC lines analysed separately in the two Tks4-KO MSCs; (**F**) Results of the gelatinase assay (repeated twice: Exp. 1 and Exp. 2) showing the degradation activity of the WT MSC line and the average of the two Tks4-KO MSC lines. The figure shows the quantitation of the relative degradation area (dark area of the FITC-gelatine surface) per cell area (TRITC-Phalloidin labelling). Student’s unpaired *t*-test was used for the statistical analysis. * *p* < 0.005.

It has already been reported that Tks4 plays a role in mouse bone marrow mesenchymal stem/stromal cell differentiation, and Tks4 mutation leads to reduced osteoblastic and adipogenic cell production. To test whether Tks4 also has an effect in human embryonic stem cell-derived MSC-like cells, we forced WT cells and Tks4-KO HUES9 clones to differentiate into MSCs (as described previously in [20]). Within 6 weeks of differentiation, both WT and Tks4-KO HUES9 cells acquired MSC-like cell morphology (Supplementary Figure S3A) and became positive for several mesenchymal stem cell markers (CD73, CD44, CD90) and negative for hematopoietic stem cell markers (CD34, CD133), pluripotency marker (TRA-1-81), and hematopoietic cell marker (CD45) as detected via FACS (Figure 3A and Figure S3B). Western blot analysis showed that expression of the EMT markers (Vimentin and Fibronectin) started uniformly in MSC cells independent of the presence or absence of Tks4 (Figure 3C).

After we were convinced that Tks4 was also present in the generated MSC-like cells (Figure 3B), we used a proteome profiler stem cell array (R&D Systems, ARY010) to study the effect of Tks4 mutation on early commitment of MSCs. As shown on Figure 3D, which highlights the overall pattern of stem cell marker expression, the original (WT and Tks4-KO) HUES9 cells expressed high levels of the Oct3./4, Sox2, and Nanog stem cell markers, but the expression of cell commitment marker proteins was under the limit of detection. In the MSC lines, pluripotency marker expression decreased and expression of proteins that regulate cell commitment started to increase.

Focusing on MSCs, we observed significant enrichment for the downregulation of cell commitment-related markers in the Tks4-KO MSC clones (Figure 3E). The decreased expression levels of mesoderm markers (Snail and FoxA2) in Tks4-KO MSC clones suggest that in case of the lack of Tks4 the MSC differentiation might be altered and less powerful than in the WT cells. Similarly, the levels of GATA4 and goosecoid (GSC) transcription factors, which have roles in bone tissue formation were lower in Tks4-KO MSCs than in the WT counterparts, further reinforcing the notion that lack of Tks4 slows differentiation [21,22]. These data are consistent with the observation that Tks4 has an instructive role in bone tissue formation, which might be related to the bone phenotypes of FTHS patients.

Interestingly, the levels of E-cadherin and Snail were lower in Tks4-KO MSCs (Figure 3E), suggesting that lack of Tks4 might alter the EMT process during MSC formation

The two Tks4-KO clones showed very similar phenotypes for the mentioned differentiation markers except for the expression level of the endoderm marker AFP. Tks4-KO 2 clone had a reduced AFP level compared with the Tks4-KO 1 clone and with the WT cells, which is consistent with the ICC results obtained in the spontaneous differentiation experiments.

In addition to the expression level changes of EMT regulator markers, another EMT-associated feature is the increased matrix degrading ability [23]. Therefore, we have performed a gelatinase activity measurement assay with the generated MSC clones. Figure 3F shows that both of the Tks4-KO cells presented higher matrix degrading activity compared with WT cells (see Supplementary Figure S3C also). In conclusion, we suggest that the absence of Tks4 might result in partial-EMT with altered E-cadherin and Snail expression levels and increased degrading ability in MSCs while the Vimentin and Fibronectin expression levels were unaltered.

## 3. Discussion

Many rare diseases that appear world-wide in the human population suffer from a lack of knowledge and methods for rapid diagnosis and therapeutics, due to difficulties in identifying the causative genetic mutations. FTHS is one of the exceptions, as we have adequate scientific background information about the single causative gene mutation (i.e., in the S*H3PXD2b* gene) that leads to the FTHS phenotypes [12]. Since the discovery of this monogenic disease, a moderate scientific effort has been dedicated to investigating the how the functions of Tks4, which is encoded by the *SH3PXD2b* gene, exert their effects on FTHS-affected tissues [4,5,24,25]. Three different mouse models have been generated to study the effects of Tks4 loss in vivo and these studies provided information about how Tks4 regulates bone and adipose tissue homeostasis [12,26,27]. The use of mice generally provides descriptive data, and these limited applications fail to provide information on the mechanism through which Tks4 protein regulates cell differentiation and interacts with signalling molecules in cells. Moreover, the use of experimental mouse cells is inferior to the use of human cells, and the results obtained in animal systems should be validated in human cells. Therefore, we decided to generate a suitable in vitro tool for further analysing the consequences of lack of Tks4 protein in human cells with broad differentiation ability. The use of pluripotent stem cell-based experiments and CRISPR/Cas9 method for Tks4 knock out would allow us to discover the effects of Tks4 directly in cells, to assess the molecular interactome/signalome of the Tks4 adaptor protein, and to validate the results of the mouse studies.

We have successfully generated two homozygous Tks4-KO clones and two heterozygous Tks4-KO clones in the HUES9 embryonic stem cell line with a normal karyotype. Our sequencing analysis showed that short indels (less than 15 base pairs) were formed in the mutated alleles in the generated cell lines. This finding is interesting as using the same CRISPR/Cas9 system in a cancer cell line (HCT116) resulted in a large insertion (more than 150 base pairs) in exon 2 of the *SH3PXD2b* gene, reinforcing the notion that embryonic stem cells have more robust genomic stability—due to more rigid genome integrity protecting machinery—compared with cancerous cells [28,29].

The Tks4-KO HUES9 cells showed three-germ-layer-differentiation potential similar to that of the original WT HUES9 cells in a spontaneous differentiation assay. Immunostaining for pluripotency markers (Oct4, Nanog) (Figure 2B) and FACS analysis for SSEA-4 (Figure 2A) confirmed the pluripotent state of the Tks4-KO embryonic cell lines. We observed variations in AFP expression in the two Tks4-KO cell lines (Figure 2C,D and Figure 3E) as in the second Tks4-KO clone we detected lower AFP expression at the protein and mRNA levels during spontaneous differentiation and in the MSC cultures. We have not examined the basis for this difference, although it would be interesting to study the AFP expression levels in FTHS patients and in Tks4-KO mice to potentially identify a correlation between reduced AFP expression and FTHS-associated phenotypes. AFP is critical for the proper development of gonadal tissue; therefore, lack of AFP might partially be the cause of infertility observed in *SH3PXD2b* -mutant mice and FTHS-affected individuals [30].

There was no difference in the proliferative capacity of the studied cells; however, the viable cell number varied more in the Tks4-KO clones. This difference might be consequence of Tks4 loss but needs further analysis. In summary, our results indicate that a lack of Tks4 does not interfere with basic germ layer formation. Considering that all three germ layers develop during embryogenesis in FTHS patients and Tks4-KO mice, leading to viable progeny, it is not surprising that the Tks4-KO HUES9 cells also retained broad differentiation ability. We hypothesize that lack of Tks4 could disrupt specification and presumably the full cell-differentiation process as well as resulted in altered cell maturation in vivo and in HUES9 cells. Mature bone and fat cells (two FTHS-affected cell types) are derived from a common stem cell type, i.e., MSCs. Since embryonic stem cells can form MSCs under special conditions and the MSC formation process can be followed in vitro we have challenged the MSC-developing potential of Tks4-KO HUES9 cells.

Our results demonstrated that the generated Tks4-KO cell lines can further differentiate into MSCs. Our attempts to study the effect of Tks4 loss on MSC differentiation showed that the expression levels of several mesoderm and osteogenic markers were lower in the Tks4-KO MSCs (Figure 3E). Consequently, comprehensive functional studies should be performed to further to shed light on the mechanism through which Tks4 affects stem cell differentiation.

Tks4 has been previously described as a regulator of EMT-like processes. In a study using a cancer cell system, lack of Tks4 promoted the occurrence of partial EMT, suggesting that the EMT-signalling pathways might be destabilized and disturbed. In addition to its role in cancer development and cell transformation, EMT is also important during embryonic cell differentiation as several cell invasion steps occur. Therefore, we have measured the expression levels of EMT-related factors (Fibronectin, SNAI1, Twist) during spontaneous differentiation process of HUES9 cells. In non-cancerous cells as hESC, we did not detect major differences in the expression levels of EMT factors between WT and Tks4-KO cells, and we concluded that loss of Tks4 does not disturb the EMT process in embryonic stem cells. By contrast, the newly generated Tks4-KO hMSCs showed alterations in EMT-related features, including matrix degrading ability and the expression levels of some EMT markers (E-cadherin and Snail1). We hypothesize that Tks4 is not the key regulator of EMT although it plays a role in the fine tuning of this process and that a reduction in the Tks4 level in mesenchymal stem cells could result in partial EMT. The observed EMT-like events (i.e., increased matrix degrading ability and decreased E-Cadherin and Snail protein levels) in Tks4-KO hMSCs suggest that the kinetics of the differentiation process might be affected, perhaps resulting in an altered differentiated tissue composition, which underlies FTHS.

In summary, the key outcomes of this study are the generation of an in vitro human model for studying the effects of a loss of the Tks4 protein in stem cells that have the potential to differentiate into three germ layers and into cell types affected by FTHS.

## 4. Materials and Methods

### 4.1. Cell Lines and Cell Cultures

The HUES9 embryonic stem cell line was kindly provided by Douglas Melton (HHMI). This work was performed according to ethical approvals (HuES9 NIH approval NIHhESC-09-0022 and Health Care Research Council, Human Reproduction Committee in Hungary (Egészségügyi Tudományos Tanács, Humán Reprodukciós Bizottság, ETT HRB) approval number 6681/2012-EHR. HUES9). The produced Tks4-KO Hues9 cell lines were cultured as previously described [31]. Briefly, the cells were cultured on Matrigel (Corning, Corning, NY, USA)—coated plates with mTeSR1 media (Stemcell Technologies, Vancouver, Canada) and were subcultured with Accutase (Thermo Fisher Scientific, Waltham, MA, USA).

### 4.2. In Vitro Spontaneous Differentiation via Embryoid Body Formation

First, pluripotent stem cell colonies were plated on MEF (mouse embryonic fibroblast (Millipore, Burlington, MA, USA)-coated plates. Next, collagenase (Thermo Fisher Scientific) was used to dissociate the cells (colonies stay together), which were then plated onto ultra-low attachment plates (Nalgene Nunc International, Rochester, NY, USA) to form EBs in suspension over 6 days. The EBs were then plated onto gelatine (Sigma-Aldrich, St. Louis, MO, USA) -coated plates for another 6 days for differentiation (as described in detail in [32]). During this process, the Tks4 protein level was monitored in WT cells (6 + 3 and 6 + 6 days), and the expression levels of germ layer markers were analysed after differentiation.

### 4.3. MSC Differentiation Process

Mesenchymal stem cells were produced from the embryoid bodies as described earlier [20]. Briefly, 6 day embryoid bodies were plated on gelatine-coated 24-well plates with DMEM supplemented with 10% FBS (Thermo Fisher Scientific) medium. After 6 + 19 days, the clones were first sub-cultured (trypsin (Thermo Fisher Scientific) passage in DMEM medium supplemented with 15% FBS). Twelve days later, the clones were again passaged using trypsin. Finally, upon the appearance of homogenous, flattered cells (approximately 1 month), FACS analysis was used to detect CD73, CD44, and CD90 markers.

### 4.4. CRISPR-Cas9 Genome Editing Method to Generate Tks4-KO HUES9 Cell Line

HUES9 cells were electroporated with pCMV-Cas9-GFP_*SH3PXD2B* (Sigma-Aldrich) using an Amaxa Nucleofector and the Human Stem Cell Nucleofector™ Kit (Lonza, Basel, Switzerland) with the A-23 program. The cells were then maintained on Matrigel-coated plates in mTeSR1 media. Two days after nucleofection, GFP-positive cells were sorted via FACS (Attune FACSARIA III sorter) onto a Matrigel-coated 96-well plate for clonal expansion. After this step, 22 clones were recovered for screening for Tks4-KO. The cells were genotyped with genomic DNA isolation and PCR specific for exon 2, a T7 endonuclease assay (NEB, New England Biolabs, Ipswich, MA, USA) specific for the Cas9 cleavage site, and Sanger sequencing. For genomic DNA isolation, the Phire Tissue Direct PCR Master Mix (Thermo Fisher Scientific) was used for genomic DNA isolation. The DNA fragment covering the gRNA target region was amplified using Tks4-specific primers [19].

**Figure 4:**
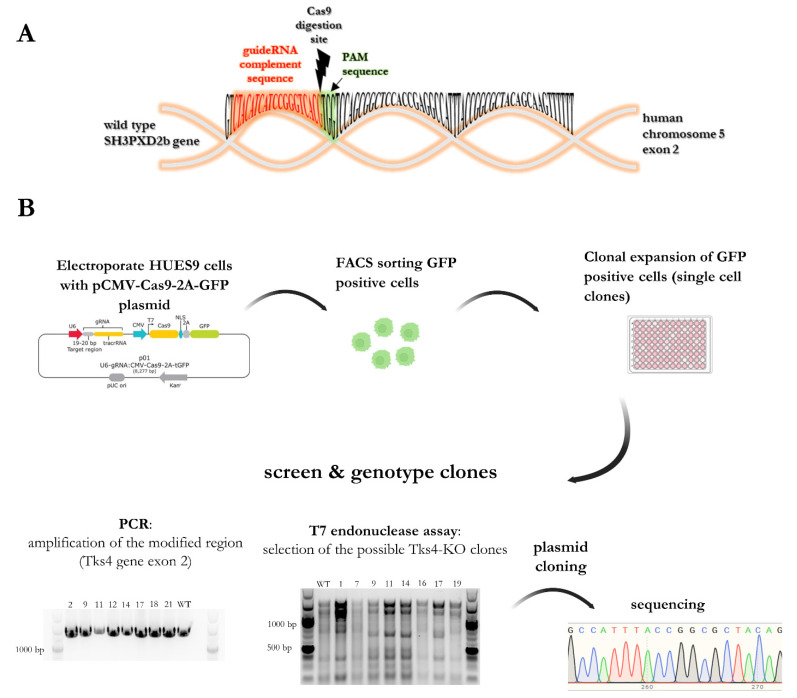
(**A**) A schematic overview of the mutant region of *SH3PXD2b* exon 2 on human chromosome 5, showing the complementary sequence of the guide RNA (gRNA), the Cas9 digestion site, and the PAM sequence; (**B**) Stepwise representation of the knock-out generation and clonal screening strategy for Tks4-KO HUES9 cells after genome editing. After single-cell plating of GFP+ cells, 21 clones were expanded for genomic DNA isolation and T7 endonuclease analysis. Next, 10 DNA samples from potentially mutated clones were used for amplification of the affected region of exon 2 and cloned in plasmid. Sanger sequencing confirmed the isolation of two homozygous Tks4-KO clones and two heterozygous Tks4 clones.

T7 endonuclease was used to detect possible KO clones and to confirm the WT clones. T7 endonuclease cuts at the site of mismatched nucleotide sequences (as occurs when Cas9 cuts and random nucleotides are incorporated, Figure 4A). In case of mismatched nucleotide sequences, T7 endonuclease produces different sized fragments compared to those generated from WT sequences (in this case, the Cas9 digestion site is in the middle of the amplified region; therefore, we expected a 700 bp fragment) (Figure 4B). A new band appearing in the T7 assay indicates that a mutation occurred due to Cas9 activity. This experiment yielded Tks4-KO candidates from among the 22 clones, and the relevant genomic sequences was cloned into a plasmid (pBluescript II SK(+) plasmids) to determine the exact type of modification. The plasmids were amplified in *Escherichia coli* DH5alpha, and plasmid DNA was isolated with the GenElute™ Plasmid Miniprep Kit (Sigma-Aldrich). The plasmid DNA was sequenced by Microsynth (Balgach, Switzerland), and the sequences were analysed in SnapGene (Chicago, IL, USA).

### 4.5. Antibodies for Western Blotting, Immunocytochemistry, and Flow Cytometry

One antibody against Tks4 was purchased from Sigma-Aldrich (HPA036471) and another polyclonal Tks4 antibody was previously generated for western blotting and immunocytochemistry (ICC) [33].

For pluripotency marker detection: Oct 3/4 antibody (Mouse anti-Oct3/4-, Santa Cruz Biotechnology (Dallas, TX, USA) Cat# sc-5279) and Nanog antibody (goat anti-Nanog, R&D Systems (Minneapolis, MN, USA) Cat# AF1997) were used for ICC. SSEA4 antibody (mouse anti-SSEA-4-PE, R&D Systems Cat# FAB1435P) and isotype control (mouse IgG3 PE-conjugated Antibody (R&D Systems Cat# IC007P) were used for flow cytometry.

For differentiation marker detection: Mouse anti-AFP antibody (Sigma-Aldrich Cat# A8452), mouse anti-SMA antibody (Abcam (Cambridge, UK) Cat# ab7817), mouse anti-ß-III-Tubulin antibody (R&D Systems Cat# MAB1195), and rabbit anti-BMP4 antibody (Abcam Cat#ab124715) were used for ICC.

For mesenchymal stem cell marker detection: Fibronectin antibody (ab45688), Vimentin antibody (ab137321), and alpha–Tubulin antibody (loading control, Abcam, ab15246) were used for western blotting. For positive MSC markers detection: PE Mouse Anti-Human CD73 (BD Pharmingen™ (Franklin Lakes, NJ, USA) Cat# 550257 and PE Mouse Anti-Human CD90 (BD Pharmingen™ Cat# 561970) with PE Mouse IgG1, κ Isotype Control (BD Pharmingen™ Cat# 559320) and FITC Mouse Anti-Human CD44 (BD Pharmingen™ Cat# 555478) with FITC Mouse IgG2b κ Isotype Control (BD Pharmingen™ Cat# 555742) were used for flow cytometry. For negative MSC markers detection: PE Mouse Anti-Human CD45 (BD Pharmingen™ Cat# 555483), PE Mouse Anti-Human CD34 (Becton Dickinson (Franklin Lakes, NJ, USA), Cat# 345802), PE Mouse Anti-Human CD133 (MACS, Miltenyi Biotec (Bergisch Gladbach, Germany), Cat# 130080801) with PE Mouse IgG Isotype Control (Becton Dickinson, Cat# 345816), Alexa 647 Mouse Anti-Human TRA-1-81 (BD Pharmingen™ Cat# 51-9006219) with Alexa 647 Mouse IgM Isotype Control (BD Pharmingen™ Cat# 51-9006225) were used for flow cytometry.

Secondary antibodies were: Goat-Anti-Mouse antibody /(H + L), Alexa Fluor 488, Thermo Fisher Scientific Cat# A-11029/ Goat anti-Rabbit IgG (H + L), Alexa Fluor 488 Thermo Fisher Scientific Cat# Cat # A-11070/ and Donkey-Anti-Goat antibody /(H + L), Alexa Fluor 488, Thermo Fisher Scientific Cat# A-11055/ for ICC analysis. An Anti-Rabbit IgG (HRP-conjugated Thermo Fisher Scientific, Cat# G-21234) secondary antibody was used for western blotting.

### 4.6. Western Blotting

For the western blot analysis, cells were harvested with lysis buffer (50 mM Hepes buffer, pH 7.4, containing 100 mM NaCl, 1% Triton X-100, 20 mM NaF, 1 mM EGTA, 1 mM Na_3_VO_4_, 1 mM p-nitrophenyl-phosphate, 10 mM benzamidine, 1 mM phenylmethylsulphonyl fluoride, 25 μg/mL each of leupeptin, soybean trypsin inhibitor, and aprotinin) and washed twice with PBS. The lysates were then centrifuged at 20,000 rpm, 4 °C, 10 min. The total protein concentration was determined using Lowry reagents, and sample loading dye (4×) was added to the supernatants followed by incubation at 100 °C for 5 min. For gel electrophoresis (SDS-PAGE), 20 ug of protein were loaded from each sample into 10% gels. Blotting was performed either at 4 °C for 1 h 300 mA or overnight at 4 °C 25 V. Nitrocellulose membranes were blocked for 60 min with 5% milk and then incubated with the appropriate antibody for 1 h at room temperature (RT) or overnight at 4 °C in 1% milk with Tween20. After several washing steps, the HRP-conjugated secondary antibody was added to the membranes. The membranes were then washed three times for 10 min. For protein detection, ECL reagents (Amersham, Little Chalfont, UK) and a ChemiDoc MP system (Bio-Rad, Hercules, CA, USA)/ X-ray films were used.

### 4.7. Immunocytochemistry and Confocal Microscopy

Cells were fixed with 4% PFA (Thermo Fisher Scientific) for 15 min at RT and then blocked and permeabilized in the same step with a complete blocking buffer containing 5% BSA, 5% FBS, and 0.1% TritonX-100 (Sigma-Aldrich) in sterile PBS for 1 h at RT. Next, cells were incubated with the appropriate antibody, and after several washing steps, secondary antibodies were used. The cell nuclei were stained with DAPI (Thermo Fisher Scientific). A Zeiss LSM-710 confocal microscopy system (Carl Zeiss microscopy GmbH, Jena, Germany) was used to detect proteins of interest with a 20× objective. Images were analysed with ZEN 3.2 software (Carl Zeiss microscopy GmbH, Jena, Germany).

### 4.8. Proteome Profiler Human Pluripotent Stem Cell Array Kit

A membrane-based antibody array (R&D system, ARY010), which can simultaneously measure the expression levels of 15 differentiation marker proteins [34,35], was used according to the manufacturer’s instructions, and the chemiluminescent signal was detected using a Chemidoc system (BioRad). ImageJ software was used to quantify the pixel densities according to the Array’s manual.

### 4.9. Invadopodia Assay

A QCM gelatine invadopodia assay was used to investigate the ECM degradation potential of the mesenchymal stem cells (Merck, Rahway, NJ, USA-ECM670). The manufacturer’s instructions were followed for the preparation of the fluorescein-labelled gelatine layer. Cells (2 × 10^5^) were seeded and incubated for 40 h. The degraded ECM appears as dark areas on the (FITC-labelled) gelatine in a HCS (high content screening) system (10× objective). The experiment was repeated twice, measuring 2–3 wells per cell line and 4–8 fields/well. ImageJ was used to analyse the results following the manufacturer’s instructions. The gelatine degradation and total cell (TRITC-phalloidin-labelled) areas were measured, and the relative degradation area was calculated. In addition to HCS measurement, representative images were acquired using a Leica DM IL Led (20× objective).

### 4.10. RT-qPCR

RNA was isolated with Trizol Reagent (Thermo Fisher Scientific) and the Zymo Pure RNA isolation kit (Zymo Research, Irvine, CA, USA). Next, the RNA concentration was measured with a Nanodrop, and 400–500 ng of total RNA was transcribed into cDNA using First strand CDNA synthesis kit for RT-PCR. Next, qPCR was performed with the relevant Taqman probes, and an Applied Biosystem Quanstudio 5 or StepOne PCR instrument was used.

### 4.11. Testing the Genetic Integrity of the Clones

Karyotyping analyses were performed by UD-GenoMed Medical Genomic Technologies, Ltd.

### 4.12. Proliferation Rate Measurements

A Bürker cell counting chamber and an automated cell counter device (Vi-Cell XR) were used to quantify cell numbers. Four wells were plated from each clone into a 96-well plate for both cell counting techniques. For the following 4 days, one well from each cell line was counted using both methods. The manual cell counting was conducted four times, and the automated cell counting was repeated two times, and the resulting data were normalized to day 1 and plotted.

### 4.13. Statistical Analyses

GraphPad Prism 8.0.1. was used to assess statistical significance using Student’s unpaired *t*-test.

## Data Availability

The data presented in this study are available on request from the corresponding authors.

## Supplementary Materials

The supporting information can be downloaded at: https://www.mdpi.com/article/10.3390/ijms23158803/s1.
