# Supplementary material for: A Novel Cell-Based Model for a Rare Disease: The Tks4-KO Human Embryonic Stem Cell Line as a Frank-Ter Haar Syndrome Model System
Source: Int J Mol Sci. 2022 Aug 8;23(15):8803. doi: 10.3390/ijms23158803 (PMC9369304; doi:10.3390/ijms23158803)
Supplement: Supplementary file 1 [file ijms-23-08803-s001.zip › ijms-1817098-supplementary.pdf]

Supplementary information

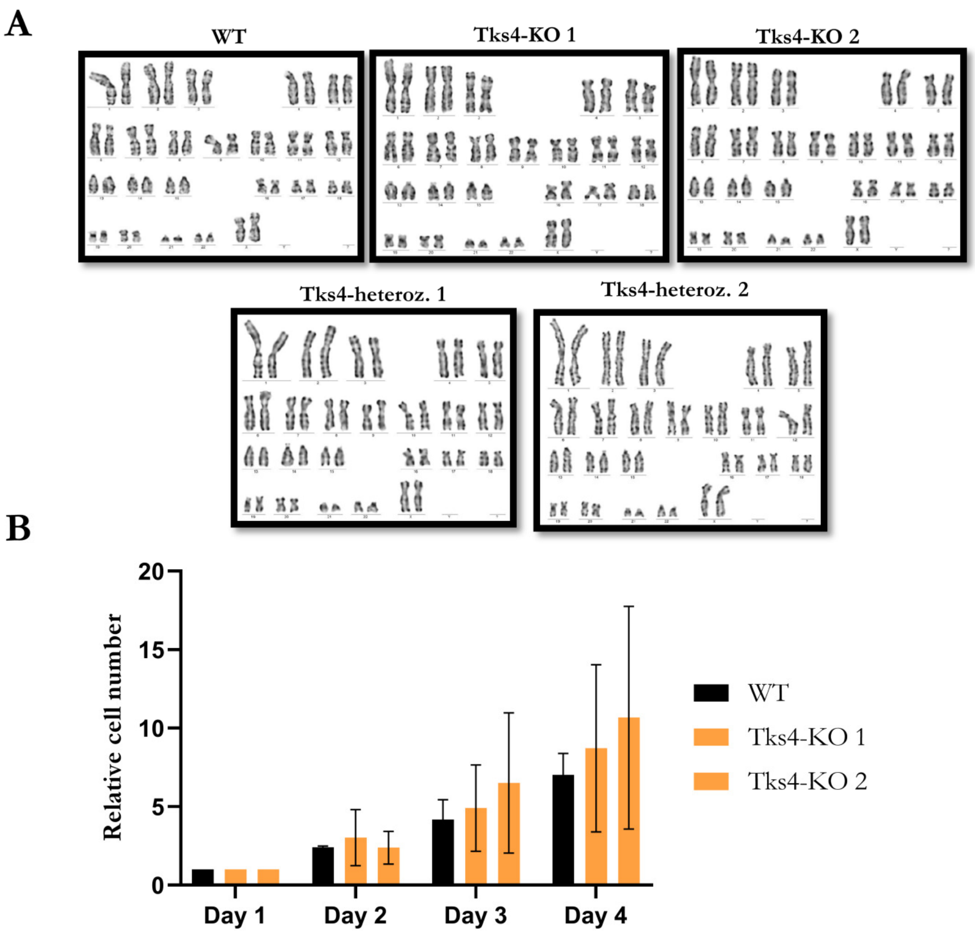

Supplementary Figure S1: (A) G-band analysis of all the used cell lines confirming a normal karyotype. (B) Proliferation rate measurements in WT and Tks4-KO clones.

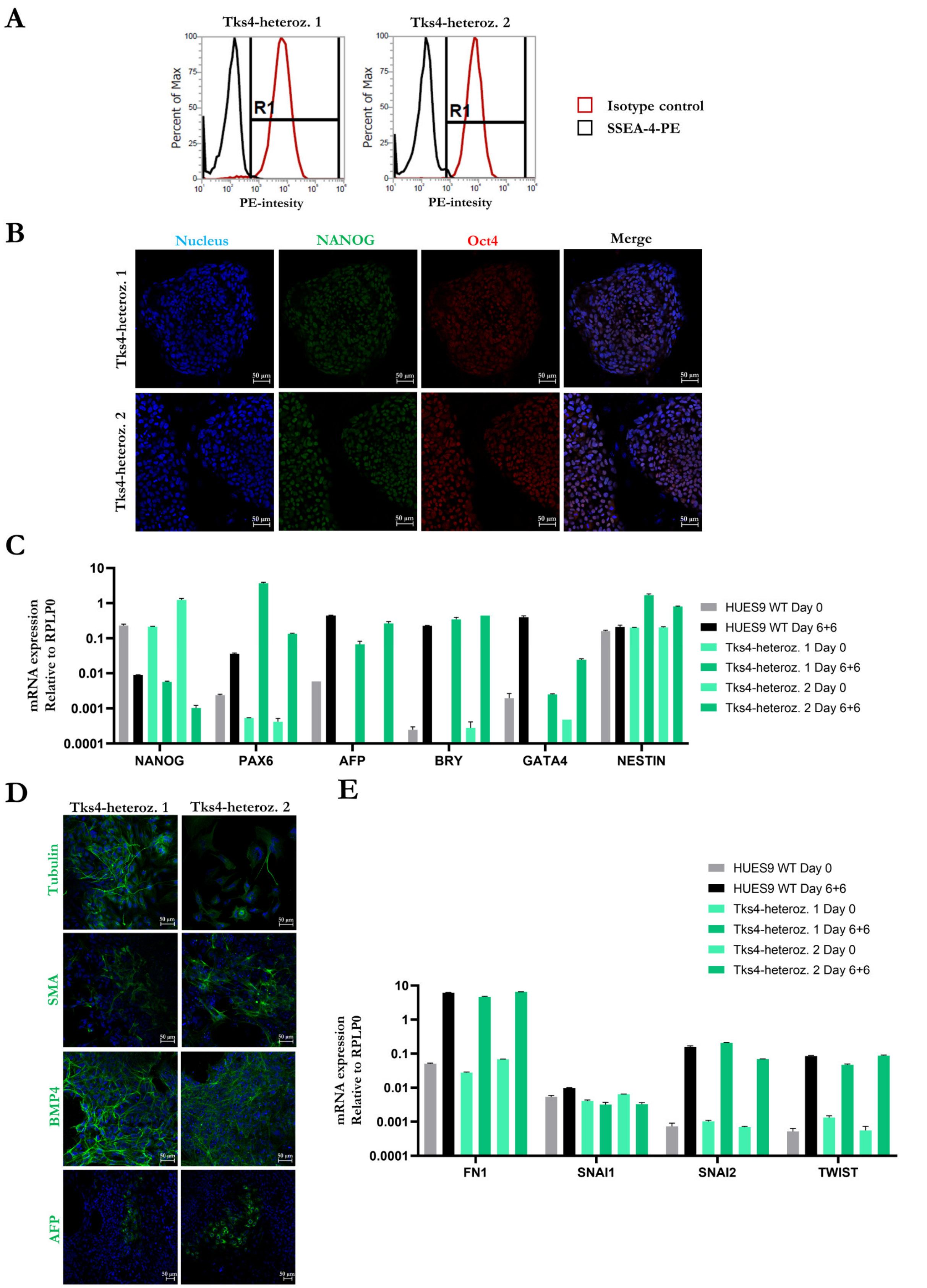

Supplementary Figure S2: Characterization of pluripotency of the established heterozygous Tks4-KO HUES9 cells. (A) SSEA4 expression by the heterozygous Tks4-KO cells was analysed via flow cytometry. Isotype controls were used as negative controls on each plot. (B)

Immunofluorescent staining showing Oct4 (red) and Nanog (green) expression in the undifferentiated cells. The nuclei were counterstained by DAPI (blue) (20x magnification). (C) The expression of pluripotency-related genes Nanog and differentiation-related Pax6, AFP, BRY, GATA4, and NESTIN were ana-lysed via RT-qPCR to assess the efficiency of EB formation. Gene expression was normalized to the value for RPLP0. (D) The three-germ-layer-differentiation potential of the heterozygous Tks4-KO cells was demonstrated by measuring the β-III-tubulin, smooth muscle actin (SMA), bone morphogenetic protein 4 (BMP4), and AFP (alpha fetoprotein) expression levels with ICC. The nu-clei were counterstained with DAPI (blue). (20x magnification) (E) Expression of EMT-related markers was detected via RT-qPCR, and their levels were compared to the original stem cell lines (day 0) within the spontaneous differentiated derivatives (day 6+6).

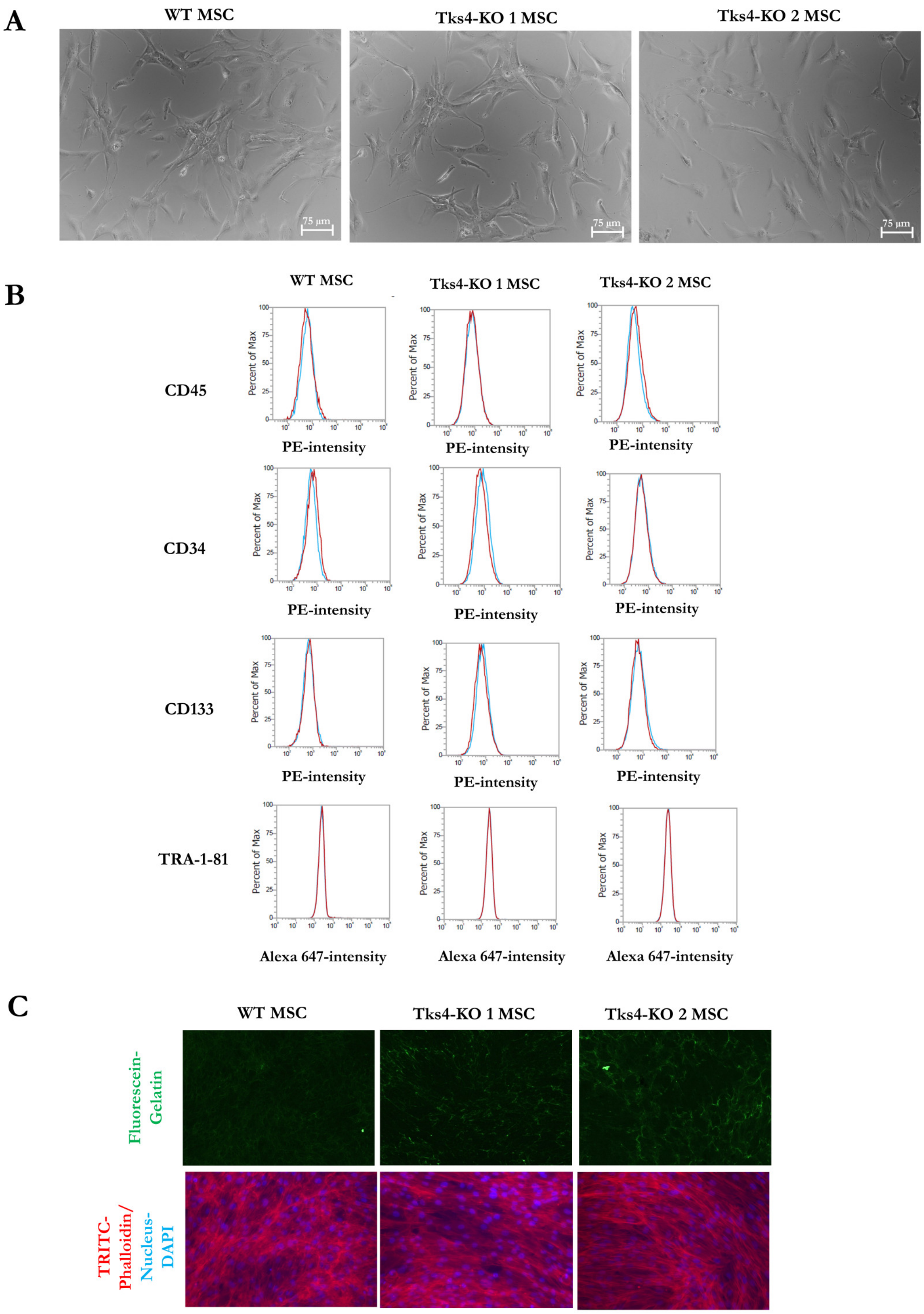

Supplementary Figure S3: (A) Representative brightfield images of the generated MSC cells showing elongated MSC-like morphology (scale bar 75 µm). (B) Representative flow cytometry histograms showing CD45, CD34, CD133, TRA-1-81 staining of WT (WT) MSC and the two Tks4-KO MSC clones. (Blue lines show the CD staining while red lines represent the isotype control la-belling) (C) Representative figures from the matrix-degradation assay, 20x objective. Fixed wells were stained with TRITC-phalloidin (red colour) and DAPI (blue colour). The presence of dark areas in the FITC-gelatine-coated culture plates indicates that the cells degraded the gelatine.
